# Effect of quercetin on muscle growth and antioxidant status of the dark sleeper *Odontobutis potamophila*

**DOI:** 10.3389/fgene.2022.938526

**Published:** 2022-07-25

**Authors:** Chenxi Zhu, Guoxing Liu, Xiankun Gu, Jiawen Yin, Aijun Xia, Mingming Han, Tongqing Zhang, Qichen Jiang

**Affiliations:** ^1^ Freshwater Fisheries Research Institute of Jiangsu Province, Nanjing, China; ^2^ College of Animal Science and Technology, Yangzhou University, Yangzhou, China; ^3^ The Lowtemperature Germplasm Bank of Important Economic Fish of Jiangsu Provincial Science and TechnologyResources (Agricultural Germplasm Resources) Coordination Service Platform, Freshwater Fisheries Research Institute of JiangsuProvince, Nanjing China; ^4^ Biology Program, School of Distance Education, Universiti Sains Malaysia, Minden, Malaysia

**Keywords:** muscle growth-related gene, quercetin, antioxidant, Odontobutis potamophila, flesh quality

## Abstract

Quercetin is a flavanol beneficial in reducing fat, promoting muscle growth, and Anti-oxidation. To study its effects in freshwater fish, the full-length cDNA of the follistatin (*FST*) and myostatin (*MSTN*) genes of the dark sleeper *Odontobutis potamophila* were cloned for the first time. Juvenile individual *O. potamophila* was exposed to quercetin at one of four concentrations (0, 2.5, 5, and 10 mg/L) for 21 days. The expression level of MSTN which inhibits muscle growth in the quercetin solution was lower than in the unexposed control group. The genes that promote muscle growth are in TGF-β superfamily like *FST*, *TGF-β1* (transforming growth factor-beta 1), and Myogenic regulatory factors (MRFs) like *Myf5* (myogenic factor 5)*, MyoD* (myogenic differentiation)*, MyoG* (myogenin), were higher than in the control group. Apolipoprotein and growth hormone receptor transcription levels in the quercetin-treated fish were significantly lower than in the control group. The concentrations of triglyceride, low-density lipoprotein cholesterol, and high-density lipoprotein cholesterol in the muscle tissue decreased, and the lipid-lowering function of quercetin was also demonstrated at the biochemical level. In this study, we analyzed the mRNA levels of *AKT*, *Keap1* (kelch-like ECH-associated protein 1), *Nrf2* (NF-E2-related factor 2) oxidation-related genes in the Nrf2/ARE antioxidant pathway, and Malondialdehyde (MDA), catalase (CAT) activity and glutathione (GSH) content in the hepatopancreas of *O. potamophila* after quercetin treatment, the mRNA expression of *AKT*, *Nrf2* and CAT activity and GSH content are higher than in the control group. Quercetin enhances antioxidant properties and positively affects muscle growth. The results showed that quercetin has no significant effects on the growth performance of *O. potamophila*, but is effective in increasing muscle growth rate and lowering muscle fat content.

## 1 Introduction

Quercetin is a flavanol widely distributed in food and vegetables such as tea, apples, cocoa, onions, and red wine (Aebi, 1984). It has been shown to have beneficial biological effects on health, including antioxidant, anti-inflammatory, anti-tumor, and anti-bacterial effects, as well as pharmacological effects on cardiovascular system protection (da Silva et al., 1998; Shutenko et al., 1999; Prince and Sathya, 2010). Quercetin can inhibit dietary energy absorption, regulate body fat metabolism, and inhibit triglyceride deposition, showing significant hypolipidemic effects both *in vivo* and *in vitro* in animals (Cai-ke et al., 2012; Pallauf et al., 2017; Forney et al., 2018; Pourteymour Fard Tabrizi et al., 2020). It ameliorates lipemia, hypertension, and hyperinsulinemia in obese rats while reducing weight gain and prolonging life span (Rivera et al., 2008). However, information on its effects on aquatic animals is quite limited.

Follistatin (FST) is a secreted protein that binds to, and inhibits, the activity of many proteins, including bone morphogenetic proteins (BMPs) and myostatin (MSTN), and growth differentiation factor-9 (GDF-9) and growth differentiation factor 11 (GDF-11) in the transforming growth factor β (TGFβ) superfamily (Funkenstein and Jakowlew, 1997; Iemura et al., 1998; Gamer et al., 1999; Amthor et al., 2002). FST is widely located in organisms and plays a role in a variety of physiological activities. A higher level of FST occurs in groupers (Family Serranidae) after consuming hydrolyzed porcine mucosa (HPM) feed, promoting muscle growth and improving meat quality (Lee and McPherron, 2001). The binding of MSTN to the activin A complex (Act RIIB) can be blocked by the activin-binding protein follistatin, suggesting that propeptide, follistatin, or other molecules can inhibit signaling through this pathway. MSTN is a member of the TGFβ superfamily and is involved in the inhibition of muscle differentiation and growth (Ferrell et al., 1999). In Zebrafish *Danio rerio* (Dong et al., 2014; Gao et al., 2016), Japanese ricefish *Oryzias latipes* (Chiang et al., 2016), and Eurasian carp *Cyprinus carpio* (Zhong et al., 2016), MSTN leads to inhibition of muscle growth. Knocking out MSTN in the red seabream *Pagrus major* using CRISPR/Cas9 increased skeletal muscle growth and the gene mutation resulted in a loss of protein function, causing an increase in the number of muscle cells and the diameter of muscle fibers, and consequent muscle overgrowth (Argilés et al., 2012). Marcelos (Aoki et al., 2010) demonstrated that all homologs of *FST,* such as *FST-288*, *FST-315,* and *FST-L3* promote muscle growth in mice. By using FST in transgenic zebrafish, MSTN in the muscles was suppressed, promoting muscles growth (Xu et al., 2003). The co-expression of FST and MSTN in the different somatic cell groups in the brain and muscles of bighead carp *Hypophthalmichthys nobilis* indicated that FST could suppress the expression of MSTN, and thus, promote their growth (Pang et al., 2018). A-I transports lipids and stabilizes the structure of plasma lipoproteins.

Upon binding to its receptor *ghra*, growth hormone (GH) affects the metabolism of carbohydrates, lipids, and proteins in animals. The *ghra* mediates a wide range of growth-related and metabolic functions, both directly and via insulin-like growth factor 1 (IGF-1) (Brooks and Waters, 2010; Waters, 2016). Quercetin has been used in animal experiments to treat oxidative damage caused by the triose phosphate/phosphate translocator (TPT) in zebrafish (Zhang et al., 2021). Oral administration of quercetin was found to lower blood glucose and normalize plasma lipid and protein profiles in rats suffering from diabetes (Ahmad et al., 2017). However, The effects of quercetin on muscle growth-related genes in freshwater fish have not been reported.

P2X7R is an ion path for ATP that can activate and induce ROS production through high levels of ATP(S) and alter the levels of oxidative stress markers (GSH, SOD, CAT, GPX, and GR) (Jiang et al., 2017), suggesting a correlation between P2X7R and oxidative stress. Quercetin alleviates oxidative stress through the P2X7R-mediated Nrf2/ARE antioxidant pathway, which further mediates the mRNA expression of PI3K, Keap1, and Nrf2 to prevent liver damage (Lee et al., 2019; Rubio-Ruiz et al., 2019; Zhao et al., 2021). In terms of the protective mechanisms, the excessive accumulation of reactive oxygen species (ROS) in fish can lead to tissue lipid peroxidation (POD), which can seriously damage cells (Valavanidis et al., 2006; Wu et al., 2017). Antioxidant enzyme systems, such as superoxide dismutase (SOD), catalase (CAT), peroxidase (GPX), and glutathione (GSH), can prevent excessive ROS (Lu et al., 2019) from potentially damaging tissues. High levels of ROS can interact with lipids and proteins and induce oxidative stress (Martínez-Álvarez et al., 2005). Importantly, fish muscle tissue is more sensitive to oxidative stress due to high levels of polyunsaturated fatty acids (Tokur and Korkmaz, 2007). The decrease in fish flesh quality may be related to the disruption of muscle structural integrity due to oxidative damage in fish (Buckley et al., 1995). In this study, we verified the antioxidant properties of quercetin by analyzing the mRNA levels of *AKT, Keap1, and Nrf2* oxidation-related genes in the Nrf2/ARE antioxidant pathways and the changes of antioxidant enzymes MDA, CAT activity, and nonenzymatic substance GSH content after quercetin treatment.

The dark sleeper *Odontobutis potamophila* is a commercially valuable freshwater fish that is widely distributed in the river systems of China and Southeast Asian countries (Hou et al., 2014)*,* and shows significant sexual dimorphism in growth, with males growing more than 30% faster than females over the same period (Zhao et al., 2017). Aquaculture of this species is of interest because of its high meat content, taste, nutritional value, and potentially high profitability (Wang et al., 2017; Jia et al., 2021). Therefore, *O. potamophila* was selected as the study species for this investigation of the molecular mechanism of quercetin effects in fish*.*

The aims of this study were: 1) to clone the full-length cDNA of the *FST* and *MSTN* genes; 2) to test the effect of quercetin on the growth of *O. potamophila*; 3) to examine the mRNA expression of the *FST*, *MSTN*, *A-I*, *ghra,* genes in different tissues*,* and the *Myf5, MyoD, MyoG, AKT*, *Keap1*, *Nrf2* and *TGF-β1* in muscle; and 4) to detect the effects of quercetin on growth-related and biochemical parameters and antioxidant enzyme activity in *O. potamophila*. The results of this study will provide insight into the mechanisms by which growth-related genes regulate muscle development in *O. potamophila* under various quercetin treatments at the molecular level, and extend the use of quercetin in fish culture.

## 2 Materials and methods

### 2.1 Ethics statement

This article does not contain any studies with human participants by any of the authors. All applicable international, national, and/or institutional guidelines for the care and use of animals were followed.

### 2.2 Animal culture

The *O. potamophila* were obtained from the Freshwater Fisheries Research Institute of Jiangsu Province, Nanjing, China. The 144 *O. potamophila* used in this study had an individual weight of 1.1 ± 0.05 g. The temperature and pH of the water were maintained at 25 ± 1°C and 7.2 ± 0.2, respectively. The dissolved oxygen concentration in the water was maintained at about 5.0 mg L^−1^. During the acclimation period, all were fed a diet of *Limnodrilus hoffmeisteri*. The feeding rate was set at 5% of the fish body weight. Quercetin solution was completely changed every 2 days to ensure stable experimental concentration.

### 2.3 Experimental design and sample collection

After acclimation, the fish were not fed for 24 h before being exposed to the different experimental quercetin concentrations (0, 2.5, 5, and 10 mg/L) for 21 d. Judging by preliminary experiments, 10 mg/L of quercetin was not harmful to *O. potamophila*. Quercetin (Sigma-Aldrich), purity >98%, was dissolved in dimethyl sulfoxide before use and stored at −20°C in the dark. There were six replicate aquariums for each treatment condition and each aquarium contained six *O. potamophila,* all living under the same cultural conditions.

During the experiments, all of the fish were fed *L. hoffmeisteri* twice daily (at 7:00 a.m. and 8:00 p.m.). The feeding rate was set at 5% of the fish body weight. Individuals were selected and treated with different concentrations of quercetin (0, 2.5, 5, and 10 mg/L) for 21 d. The quercetin solution was changed every day. Weight data were recorded at 7 d, 14 d, and 21 d during the experiment.

After 3 weeks, 144 sample fishes were anesthetized over ice, and samples of muscle tissue, gill tissue, intestinal tissue, and hepatopancreas tissue were collected using sterile scissors and forceps. The tissue samples were stored in liquid nitrogen. A control group without quercetin exposure was used for comparison and the gene cloning experiments.

### 2.4 RNA extraction and full-length cDNA cloning

TRIzol reagent (Aidlab Biotech Co., Beijing, China) was used to extract the total RNA for differential gene expression, according to the manufacturer’s instructions. *FST* and *MSTN* gene fragment data were obtained from the *O. potamophila* genome database or existing transcriptome data and analyzed by comparing their open reading frames (ORFs). Primers for the relevant genes were designed using Primer Premier 5.0. The names and sequences of the primers used are shown in Table 1. Full-length fluorescent quantitative primers *FST* F, FST R, *MSTN* F, *MSTN* R, *ghra* F, *ghra* R, *A-1* F, *A-1* R, *AKT* F, *AKT* R, *Keap1* F, *Keap1* R, *Nrf2* F, *Nrf2* R, *Myf5* F, *Myf5* R, *MyoD* F, *MyoD* R, *MyoG* F, *MyoG* R, *TGF*-β1 F, *TGF*-β1 R. (Table 1). and *O. potamophila* β-actin gene-specific upstream and downstream primers β-actin F, β-actin R were designed as internal reference genes. The RACE PCR primers used in this paper are also shown in Table 1.

**TABLE 1:** Primer names and sequences.

Primer name	Amplification efficiency (%)	Sequence (5′to 3′)	Amplicon sizeL (bp)
qPCR -*FST* F	92.3	ACT​CGG​ACT​ACA​CGG​CCT​AT	77
qPCR -*FST* R	92.3	AGA​ACT​GTC​CCC​GTA​TTG​CG	77
qPCR -*MSTN* F	94.9	CGG​ACA​AGA​TGC​CTG​TGA​GT	88
qPCR -*MSTN* R	94.9	TGT​GTG​TCC​TGT​TCA​CCG​AG	88
qPCR- *APOA1* F	93.5	GGA​TCT​GCG​CAC​CTC​TAT​CC	101
qPCR- *APOA1* R	93.5	GGA​TCT​GCG​CAC​CTC​TAT​CC	101
qPCR-*ghra* F	94.1	AGC​CAG​AGC​GTA​GCA​AAC​TT	156
qPCR-*ghra* R	94.1	GTT​GGG​GGT​GAG​TAA​GAG​GC	156
qPCR-*AKT* F	93.5	CCG​AGA​TTG​TCT​CCG​CTC​TC	170
qPCR-*AKT* R	93.5	GGA​CAC​CAC​TTG​GTC​TCT​CG	170
qPCR-*Keap1* F	94.2	CGT​GGG​TGT​AGC​CAT​TAC​CA	144
qPCR-*Keap1R*	94.2	TGA​CTG​TGC​TGC​TGA​CTC​TG	144
qPCR-*Nrf2* F	92.6	GCC​AAT​CAC​TAA​TGC​GGC​AG	80
qPCR-*Nrf2* R	92.6	GCC​ACT​GTT​GTA​GCC​ACT​CT	80
qPCR-*Myf5* F	92.7	AGG​GAC​TCC​TCT​CGT​GCA​TT	146
qPCR-*Myf5* R	92.7	CTC​CAT​GCC​AGG​ACC​AAA​GT	146
qPCR-*MyoD1* F	92.3	ACG​CCA​TCA​GCT​ACA​TCG​AG	157
qPCR-*MyoD1* R	92.3	GTA​ACA​GGT​GTC​CGC​TCA​CT	157
qPCR-*MyoG* F	95.1	GGT​GTC​CTC​CCT​AAA​CCA​GC	130
qPCR-*MyoG* R	95.1	CCG​AAC​TAG​GCT​CAC​TCG​AC	130
qPCR-*TGF*-β1 F	95.0	GTG​GGG​AAA​TCT​GCC​CGT​TA	147
qPCR-*TGF*-β1 R	95.0	CAG​CCG​AAG​TTG​GAA​GAC​CT	147
Beta-actin F	92.2	CTC​TTC​CAG​CCA​TCC​TTC​CT	220
Beta-actin R	92.2	TCA​GGT​GGG​GCA​ATG​ATC​TT	220
5′RACE-*FST* F1	—	TTGCCAGCTTGAACTT	-
5′RACE-*FST* F2	—	TGATGTTCCATAAGGTGA	—
5′RACE-*FST* F3	—	AATGCCCGGGTGGAGGTG	—
3′RACE-*FST* R1	—	GTC​CAG​AGA​GCC​GAA​CAG​ATG​AGG	—
3′RACE-*FST* R2	—	GCG​TGT​TCA​ATG​GGA​GTT​CTG​CTG	—
5′RACE-*MSTN* F1	—	GCAGGAACACCGTGGT	—
5′RACE-*MSTN* F2	—	GCAGGTGAACCCACAGCT	—
5′RACE-*MSTN* F3	—	TTGACTCGGCTGGAACTT	—
3′RACE-*MSTN* R1	—	AGC​GTT​ACA​AGG​CCA​ACT​ACT​GCT	—
3′RACE-*MSTN* R2	—	TGC​AGA​AGT​ACC​CAC​ACA​CTC​ACC	—

First-strand synthesis of cDNA was performed using a RevertAid First Strand cDNA Synthesis Kit (Fermentas, Burlington, Canada). cDNA was synthesized for gene cloning using the PrimeScript RT reagent kit (Takara, Shinga, Japan). Full-length sequences of cDNAs for *FST* and *MSTN* were obtained according to the instructions for the SMARTer RACE5 '/3′ kit, and the expression products were negative. In order to check the sharpness of bands and fragment lengths, the PCR products of amplified *FST* and *MSTN* were examined using gel electrophoresis on an agarose matrix.

The MSTN and FST PCR products with clear, accurate bands were sent for sequencing (by Baibaxun Biotechnology Co., Shanghai, China). The validity of the cDNA sequences was checked by comparing the sequencing results with the amino acid sequences of the same genes in the NCBI database, using BLASTP. Following completion of the comparisons, the 5 and 3′ ends were obtained, and the full-length *FST* and *MSTN* sequences were obtained by splicing the ends and intermediate sequences using the DNAMan software.

### 2.5 Bioinformatics analysis

In this study, ORF intervals were predicted using the NCBI ORF Finder. Homologous proteins were retrieved and analyzed using BLASTP, and protein hydrophobic regions were analyzed using ProtScale on the ExPASy server. ProtParam on the ExPASy server was used to calculate amino acid compositions, relative molecular weights, and isoelectric points. The signal peptides were predicted using the SignalP 5.0 Server from DTU Health Tech (Lyngby, Denmark). Multiple sequence alignment was performed using seeded guide trees and HMM profile-profile techniques using Clustal Omega from EMBL-EBI (Hinxton, United Kingdom). Protein structural domains were analyzed using SMART, and secondary and tertiary structures were analyzed using the PSIRED Protein Structure Prediction Server and the SWISS-MODEL Server, respectively. Sequences were aligned with MAFFT (Katoh and Standley, 2013) using “--auto” strategy and normal alignment mode. Gap sites were removed with trimAl (Katoh and Standley, 2013) using “-strictplus” command. ModelFinder (Katoh and Standley, 2013)was used to select the best-fit model using BIC criterion. Maximum likelihood phylogenies were inferred using IQ-TREE (Nguyen et al., 2015) under the JTTDCMut + G4 model for 20,000 ultrafast (Minh et al., 2013) bootstraps, as well as the Shimodaira–Hasegawa–like approximate likelihood-ratio test (Minh et al., 2013).

### 2.6 Quantitative real-time PCR analysis

The RNA extracted from the hepatopancreas, muscle, gill, and intestinal tissues of *O. potamophila* was reverse transcribed into cDNA using a PrimeScript RT reagent Kit (Takara, Shinga, Japan) and stored at −80° for real-time fluorescence qPCR analysis. Total RNA from each tissue sample was analyzed by RT-qPCR using CFX96 RT-PCR (BioRad, Hercules, CA, United States) and TransStart Top Green qPCR SuperMix (TransGen, Beijing, China).

### 2.7 Measurement of antioxidant enzyme activity and biochemical indicators in muscle

Triglycerides (TGs), low-density lipoprotein cholesterol (LDL-C), and high-density lipoprotein cholesterol (HDL-C) levels were measured using a kit supplied by the Nanjing Jiancheng Bioengineering Research Institute (Nanjing, China). The TG content was calculated by mixing 10 μl of tissue homogenate with 1 L of enzyme agent, incubating at 37°C for 10 min, and then measuring the color at 510 nm colorimetrically. The resulting H_2_O_2_ reacted with 4-AAP to produce a red-purple pigment, and the LDL-C and HDL-C contents were tested by absorbance at 546 nm. GSH in tissues can be measured colorimetrically at 405 nm. CAT activity was measured by monitoring the stable complex produced by H_2_O_2_ with ammonium molybdate, measured with ammonium molybdate at 405 nm optical diameter. MDA in peroxidized lipid degradation products was measured calorimetrically at 405 nm.

### 2.8 Data analysis

The experimental data were examined graphically using GraphPad Prism 8 (GraphPad Software, San Diego, CA, United States) and SPSS 20.0 software (IBM, Armonk, NY, United States). The relative mRNA levels of target genes were analyzed using the 2^−ΔΔCt^ method (Livak and Schmittgen, 2001). One-way analysis of variance (ANOVA) was used to test the significance of differences in growth data and muscle growth-related gene expression between the various quercetin-treated groups and the control.

## 3 Results

The datasets presented in this study can be found in online repositories. The complete mRNA sequences for *FST* and *MSTN* were submitted to GenBank with the accession numbers OK641659 and OK641660, respectively. The *FST* sequence encodes for 506 amino acids. The predicted molecular mass of the protein was 38.60 kDa with an estimated pI of 6.45. The *MSTN* sequence encodes 376 amino acids. The predicted molecular mass of the protein was 42.42 kDa and the estimated pI is 5.66.

### 3.1 Full-length sequence analysis, Amino acid sequence homology of genes

**FIGURE 1:**
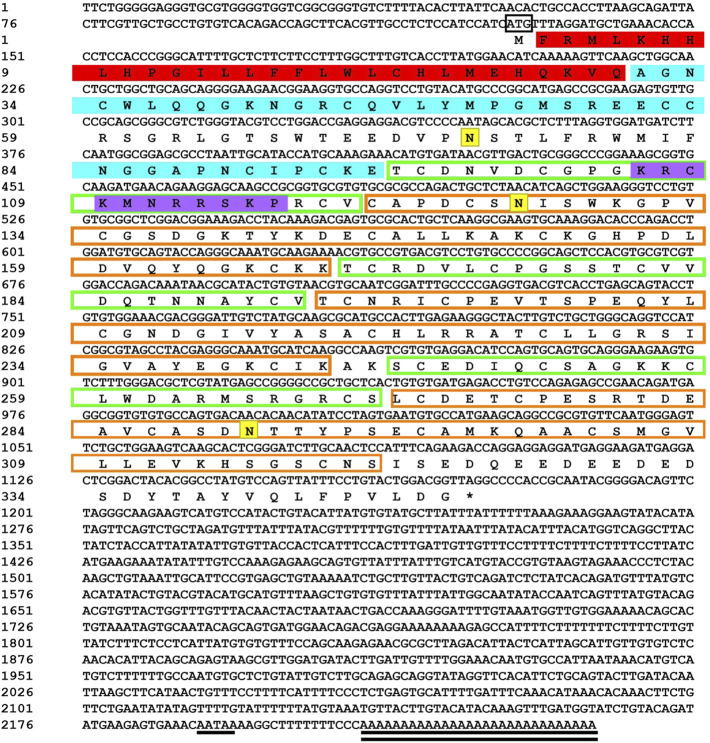
Sequencing results and amino acid sequence analysis of transcription factor FST of Odontobutis potamophila. The black box indicates the ATG start codon; * indicates the termination codon; underlines indicate the Poly(A) plus tail signal; double underlines indicate the poly(A) sequence; red shading indicates a possible signal peptide region; blue shading indicates a possible TGFβ binding structural domain; green boxes indicate three possible repetitive follistatin structural domains; orange boxes indicate three duplicated Kazal structural domains; purple shading indicates possible nuclear localization signals; and yellow shading indicates possible N-glycosylation sites.

**FIGURE 2:**
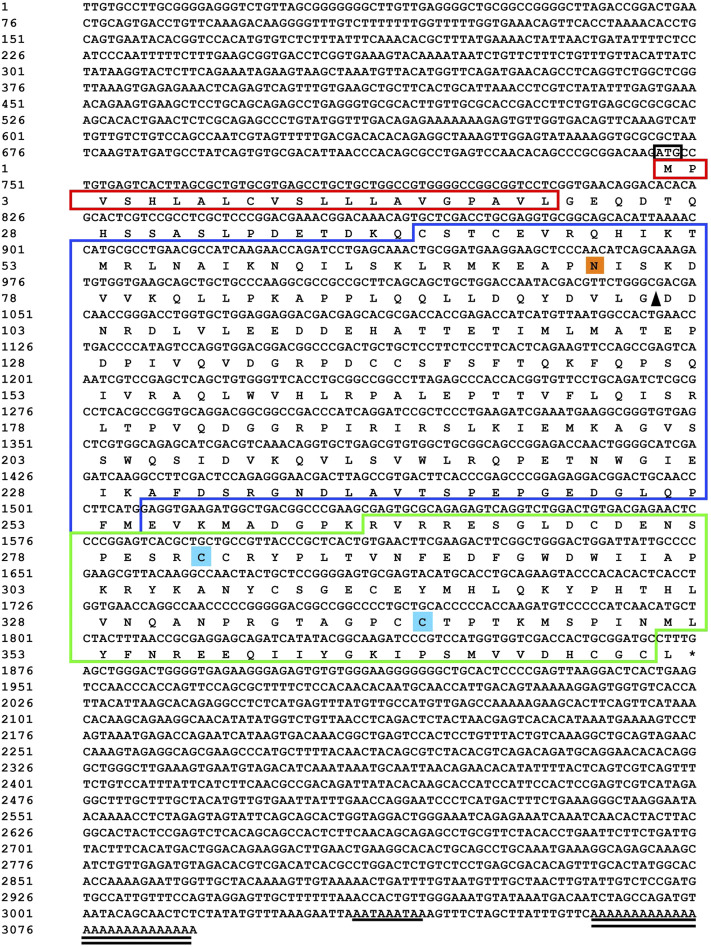
Sequencing results and amino acid sequence analysis of MSTN in Odontobutis potamophila. The black box indicates the ATG start codon; * indicates the termination codon; underlines indicate the Poly(A) plus tail signal; double underlines indicate the poly(A) sequence; red shading indicates possible signal peptide regions; blue shading indicates possible TGFβ binding structural domains; green boxes indicate three possible repetitive follistatin structural domains; orange boxes indicate three duplicated Kazal structural domains; purple shading indicates possible nuclear localization signals; and yellow shading indicates possible N-glycosylation sites.

**FIGURE 3:**
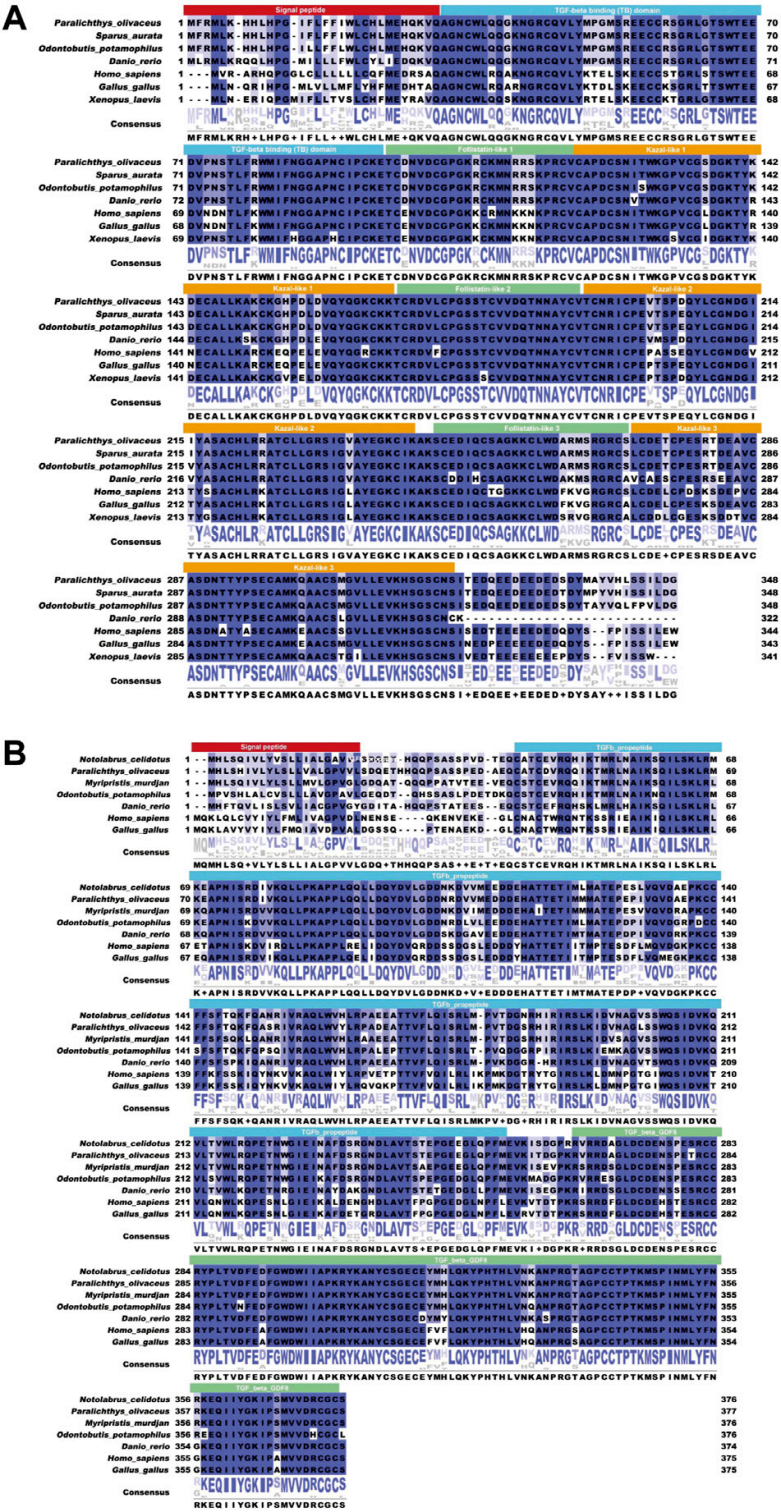
The results of the similarity analysis. **(A)** FST: the solid red box shows the signal peptide; the solid blue box shows the TGFβ binding domain; the solid yellow box shows the kazoo-like 2; the solid green box shows the FST-like 3. **(B)** MSTN: the solid red box shows the signal peptide; the solid blue box shows the TGFβ binding domain; and the solid green box shows the TGFβ GDF8.

Sequencing results and amino acid sequence analysis of transcription factor *FST* (Figure 1) and *MSTN* (Figure 2) of *O. potamophila*. The homology comparison results showed that the similarity of the *FST* sequences was very high, with the highest homology between the *O. potamophila FST* sequence and that of *Sparus aurata*, followed by *Paralichthys olivaceus.* The signal peptide region, TB (TGFβ-binding structural domain), three repeats in the *FST* of fish, mammals, amphibians, and birds, the follistatin structural domain, and three duplicated Kazal structural domains were very similar. The homology comparison results showed that the similarity between the corresponding *MSTN* sequences was very low in the signal peptide region, high in the pre-peptide structural domain, and very conserved in the C-terminal GDF8 structural domain (Figure 3).

**FIGURE 4:**
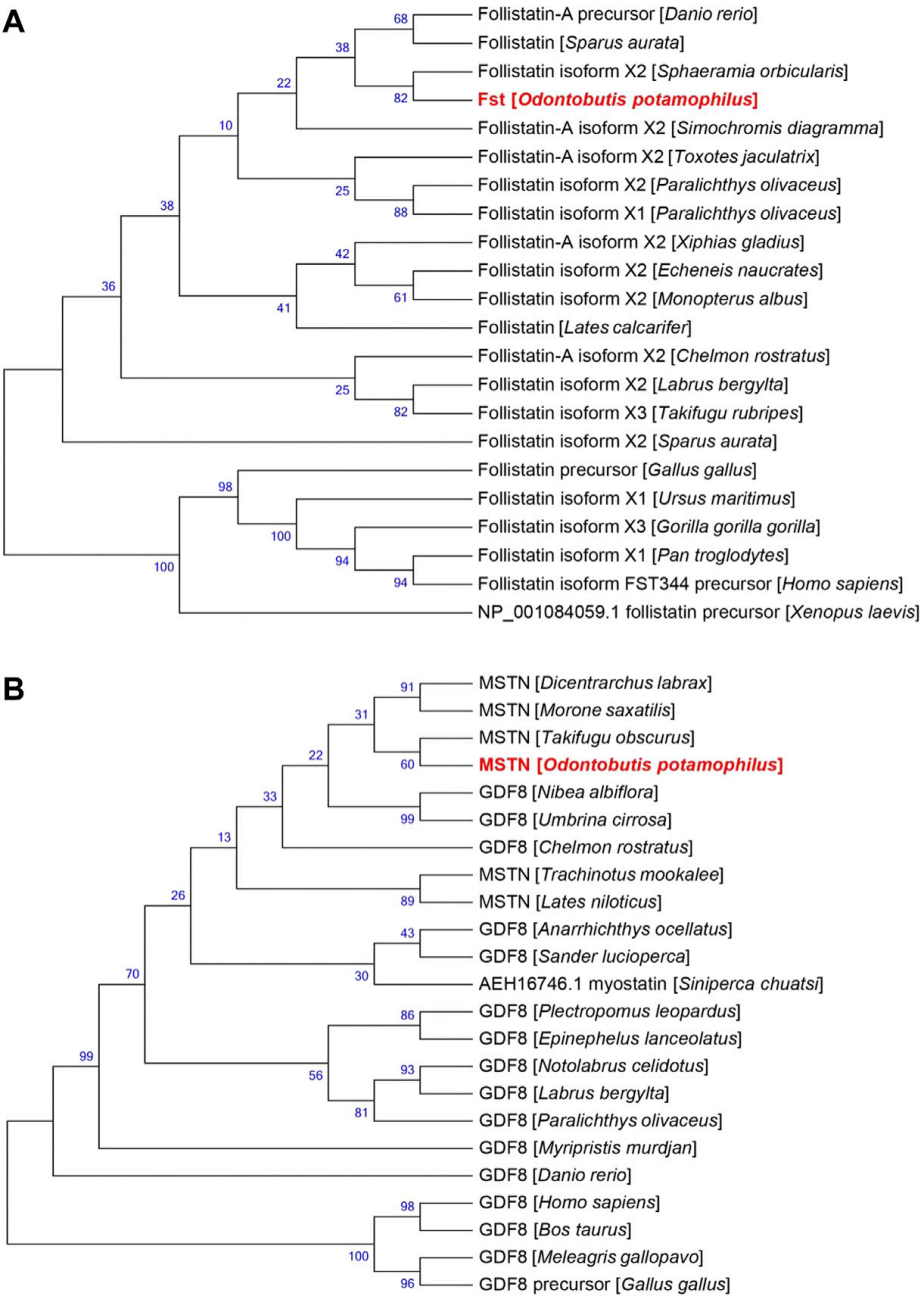
Evolutionary relationships of **(A)** FST and **(B)** MSTN in different species.

The results of the *FST* evolutionary tree comparison are shown in Figure 4A. The total length of the evolutionary tree score was 0.60789282, and the *FST* sequences of each species were relatively close, indicating a very high homology in the sequences. *FSTO. potamophilaFST*. The *MSTN* evolutionary tree comparison showed that the total length of the evolutionary tree score was 1.08964954 and that the *MSTN* sequences of each species were not very homologous. *Notolabrus celidotus*. The *MSTN* and *FST* sequences of *O. potamophila* clustered together with those of other fishes and split into two large branches in the evolutionary tree, with a split between fishes, birds, and mammals (Figure 4AB).

### 3.2 Spatial structure predictions

**FIGURE 5:**
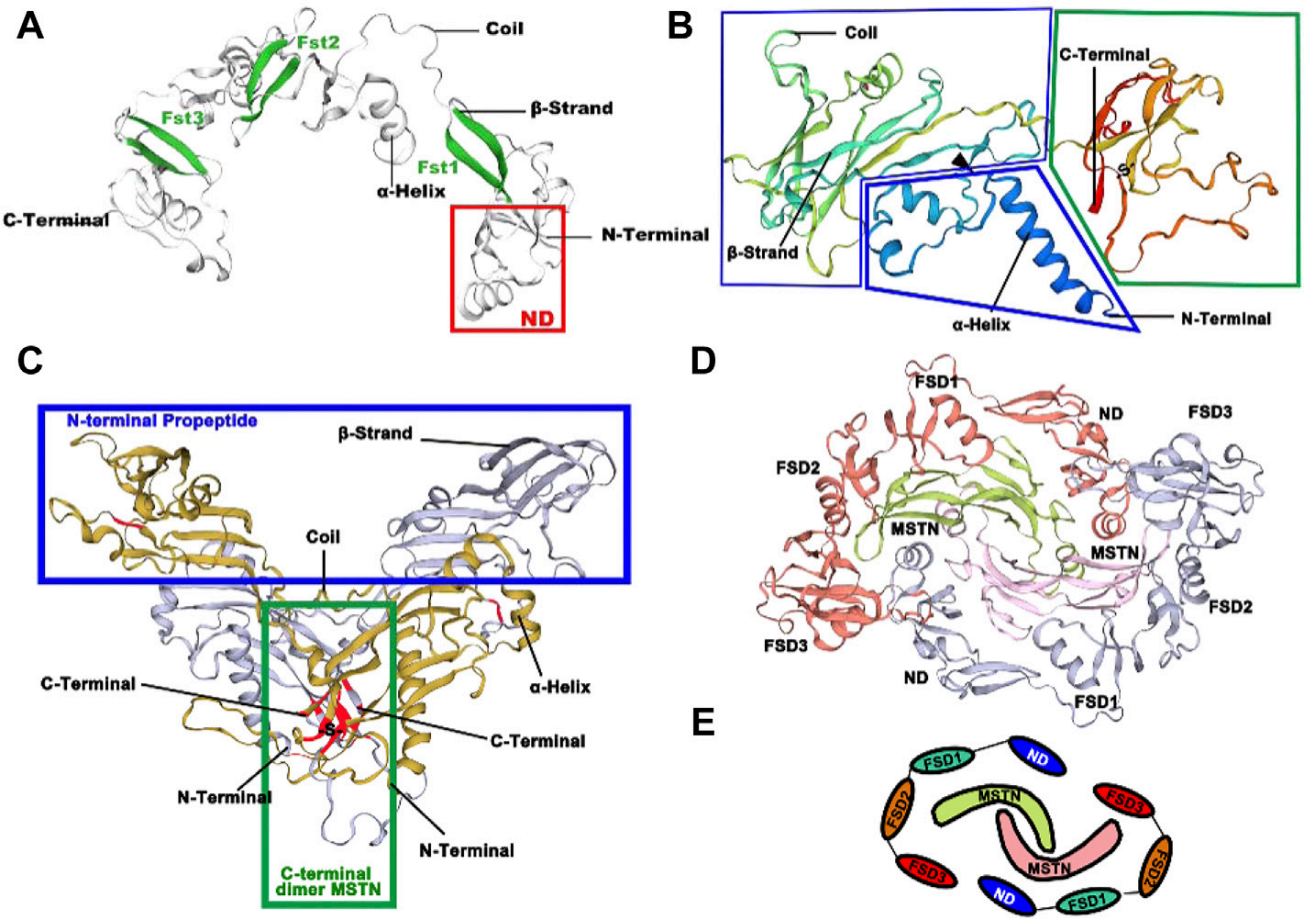
Three-dimension structures of FST and MSTN in *O. potamophila*. **(A)** Predicted 3D protein structure of transcription factor FST. **(B)** Predicted 3D protein structure of MSTN. **(C)** Predicted 3D protein structure of the MSTN homodimer. **(D)** Predicted 3D protein structure of the MSTN homodimer and the FST homodimer. **(E)** Schematic representation of the antagonistic relationship between MSTN and FST.

FST is a multi-structural protein consisting of an N-terminal structural domain (labeled ND in Figure 5A for the TB structural domain) and three resultant FST structural domains (FSD1-3). FST is an antagonist, and FST-type molecules block all four MSTN receptor binding sites to suppress signaling (Figure 5A).

The possible tertiary structure of MSTN is shown in Figure 5B, where the protein is first synthesized as a large precursor molecule, which is then cleaved by protein hydrolysis to generate an N-terminal pro-peptide and a disulfide-linked C-terminal dimer. The cyclic form comprises a potential compound of the C-terminal dimer and other proteins (including its pre-peptide) that keep the C-terminal dimer in a potentially non-active state. Activation of the ligand requires further cleavage of the precursor protein by a tolylene-like metalloprotease that breaks at a pair of Asp residues (the black triangle in Figure 5B). The C-terminus forms a dimer through a disulfide bond, with the position of the Cys residue marked in red in Figure 5C. It is concentrated in the middle of the C-terminal mature peptide, both within the single subunit and between the two dimeric subunits, and has the potential to form disulfide bonds to create a stable dimer structure at the point where one of our predicted disulfide bonds occurs between Cys282-Cys341. In Figure 5D, red and gray colors represent the two FST homodimers, and the MSTN dimer is indicated in green and pink. Figure 5E shows a schematic representation of possible FST and MSTN antagonism (Cash et al., 2009).

### 3.3 The effect of quercetin on the growth performance of *Odontobutis potamophila*

**FIGURE 6:**
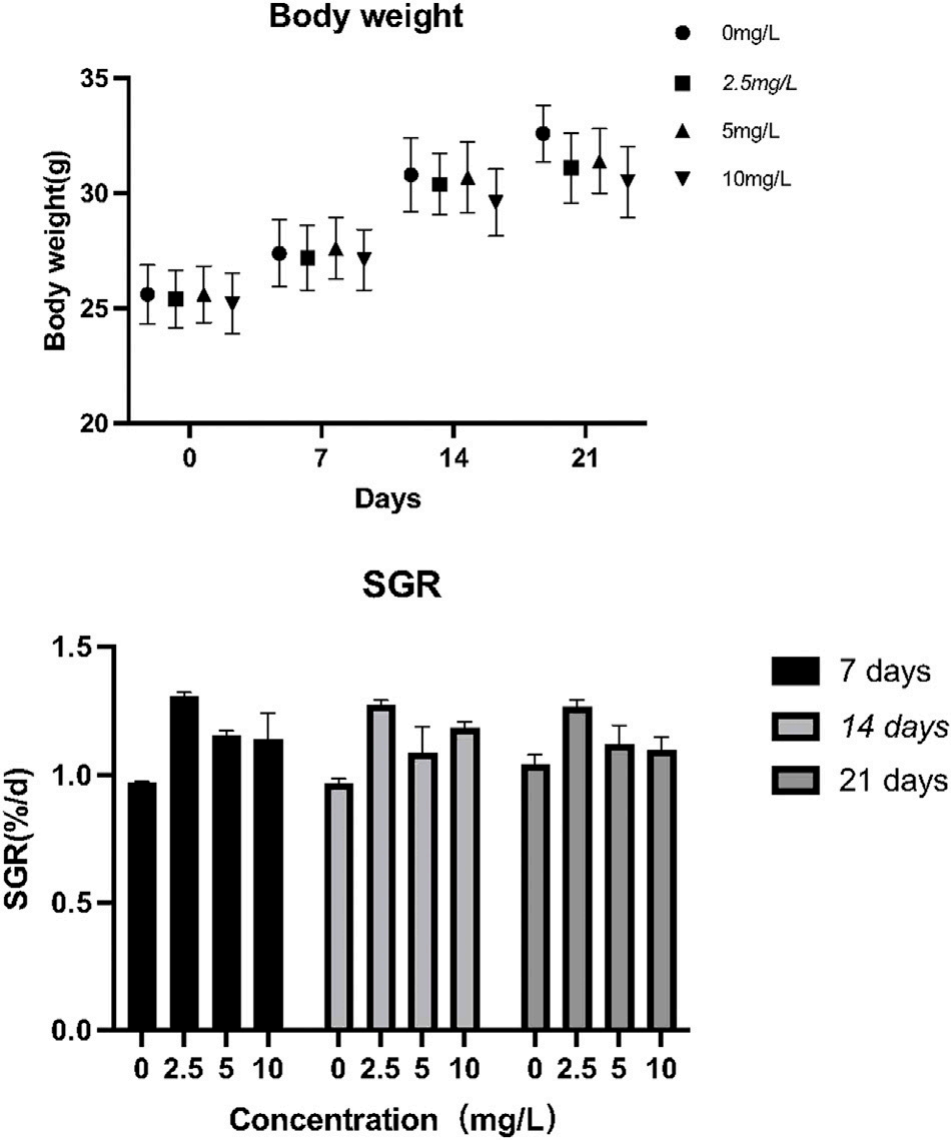
The effect of quercetin on the growth performance of *Odontobutis potamophila*. The bars indicate the mean weight ± SD (*n* = 3) of fish in the three treatment groups and the control. Statistical significance was taken as *(*p* < 0.05) and **(*p* < 0.01), compared with the control.

No significant effect of quercetin on the growth and specific growth rate of *O. potamophila* was observed in this study (Du and Turchini, 2021) (Figure 6).

**FIGURE 7:**
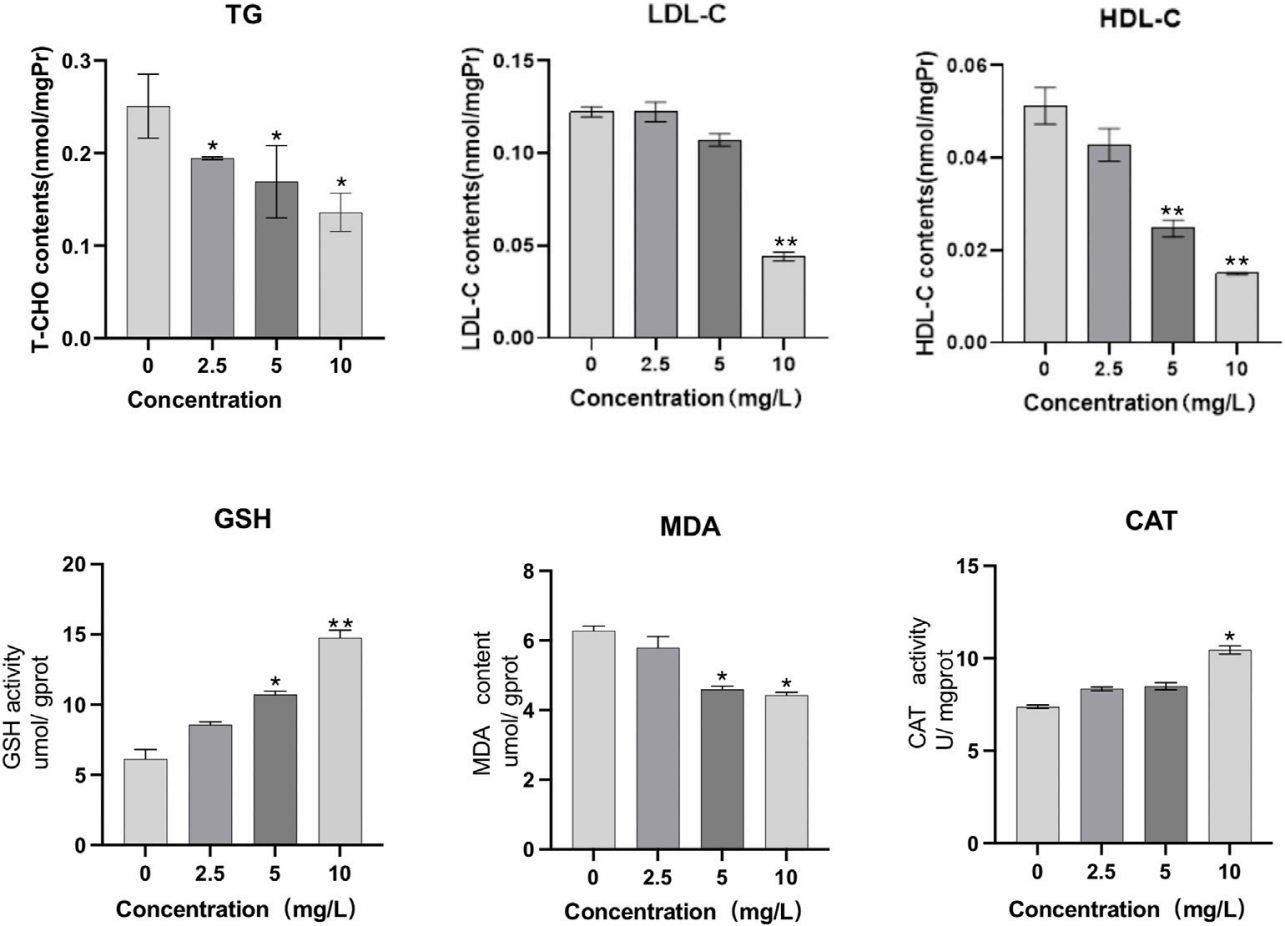
The effect of exposure to increasing concentrations of quercetin on triglyceride (TG), low-density lipoprotein cholesterol (LDL-C), high-density lipoprotein cholesterol (HDL-C), glutathione (GSH), Malondialdehyde (MDA) and catalase (CAT) in the muscles of *O. potamophila*. The bars indicate the Mean ± SD (*n* = 3). Statistical significance was taken as *(*p* < 0.05) and **(*p* < 0.01) compared with the control.

As can be seen from Figure 7, when *O. potamophila* were exposed to increasing concentrations of quercetin, a decreasing trend was observed in the muscles TG and LDL-C levels, and when the quercetin concentration was 10 mg/L, the muscle HDL-C and LDL-C levels were significantly lower than in the control group (*p* < 0.01). The activities of CAT and contents of GSH increased with increasing quercetin concentrations, with significant increases in GSH contents and CAT activities at 10 mg/L quercetin (*p* < 0.05). MDA content in the hepatopancreas of *O. potamophila* decreased as concentrations of quercetin levels increased, with significant decreases at 5 mg/L and 10 mg/L quercetin (*p* < 0.05).

### 3.4 Expression analysis of muscle growth-related genes

**FIGURE 8:**
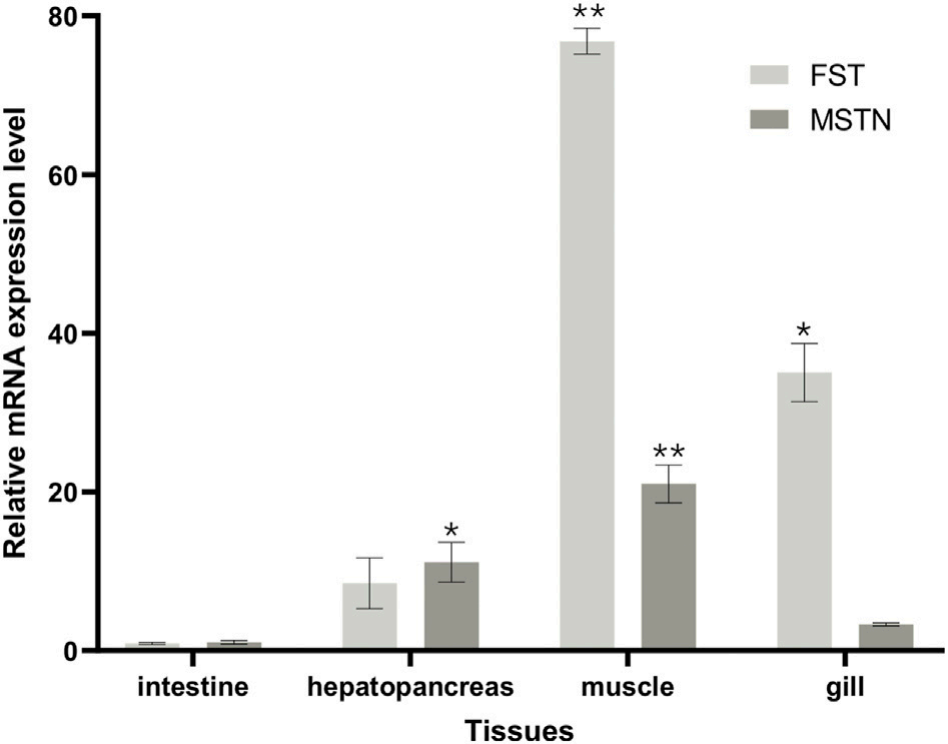
FST and MSTN gene expressions in various tissues of *O. potamophila* exposed to 0, 2.5, 5 and 10 mg/L of quercetin. The bars indicate the Mean ± SD (*n* ± 3). Statistical significance was taken as *(*p* < 0.05) and **(*p* < 0.01) compared with the intestine samples.

**FIGURE 9:**
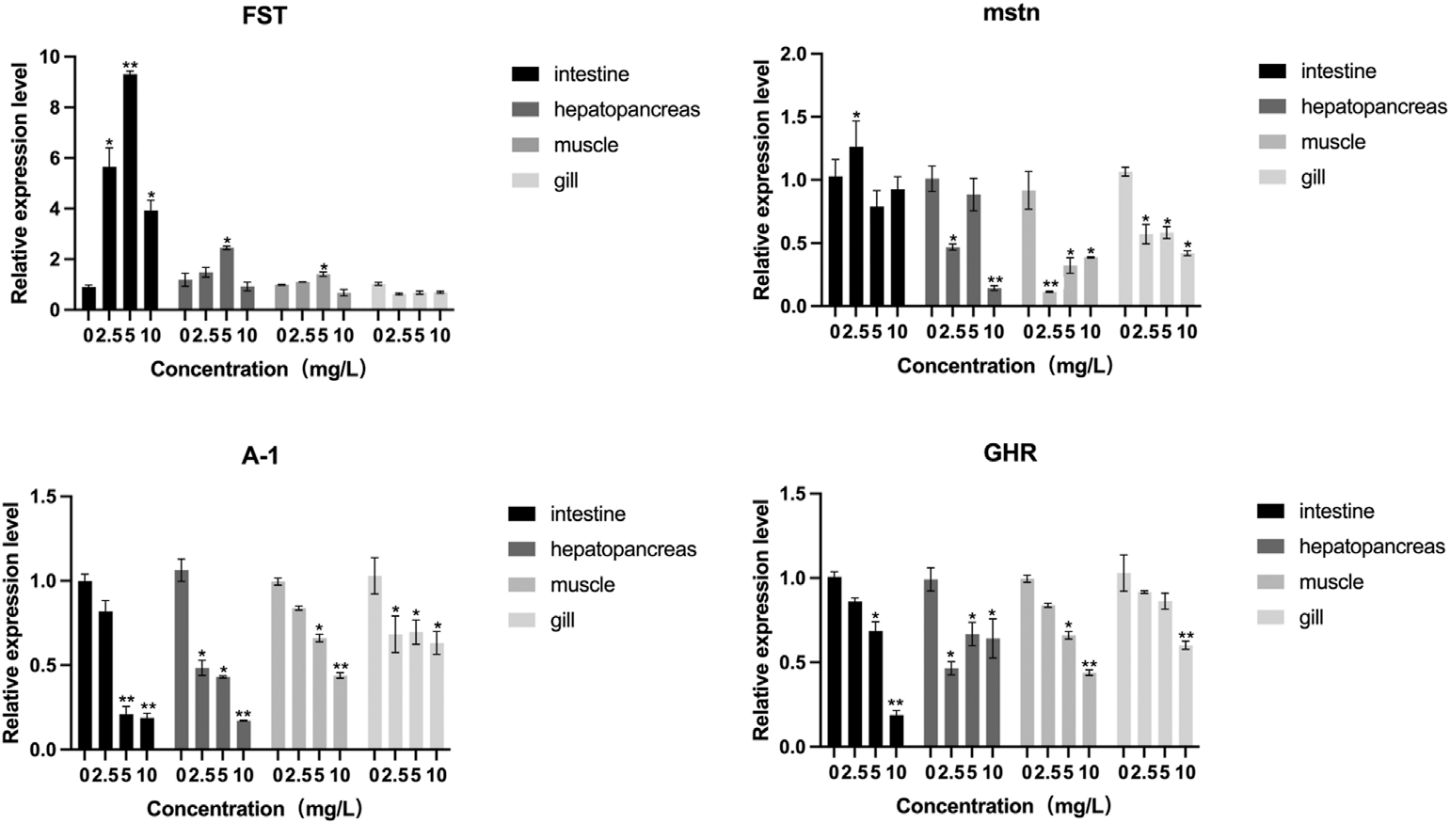
The effect of increasing concentrations of quercetin exposure on the relative expression of the FST, MSTN, A-I and GHR genes in the intestine, hepatopancreas, muscle and gill tissue of *Odontobutis potamophila* after 21 d. The bars indicate the Mean ± SD (*n* = 3). Statistical significance was taken as *(*p* < 0.05) and **(*p* < 0.01) compared with the control.

The distribution of FST and MSTN in different tissues was examined. *MSTN* and *FST* mRNA were detected in the gills, muscles, intestines, and hepatopancreas samples (Figure 9), with the highest levels found in muscle. An increasing trend of *FST* expression and a decreasing trend of *MSTN* expression in muscle and hepatopancreas tissues was found between the control and the 2.5 mg/L and 5 mg/L treatment groups. *FST* and *MSTN* were significantly expressed in muscle tissue (Figure 8). In Figure 9, the expression of the *FST* gene in the muscles of *O. potamophila* at quercetin exposure levels of 0, 2.5, and 5 mg/L increased in a stepwise manner with the highest expression in the 5 mg/L quercetin treatment group (*p <* 0.05). In contrast, the expression of *MSTN* in muscle tissue was lower in the quercetin-treated groups, and gene expression was significantly lower in the 2.5 mg/L quercetin-treated group (*p <* 0.01). Quercetin treatment reduced the expression of *A-1* and *ghra* in all tissues. In muscle tissue, *A-1* and *ghra* gene expressions were significantly reduced in the 10 mg/L treatment group (*p <* 0.01).

**FIGURE 10:**
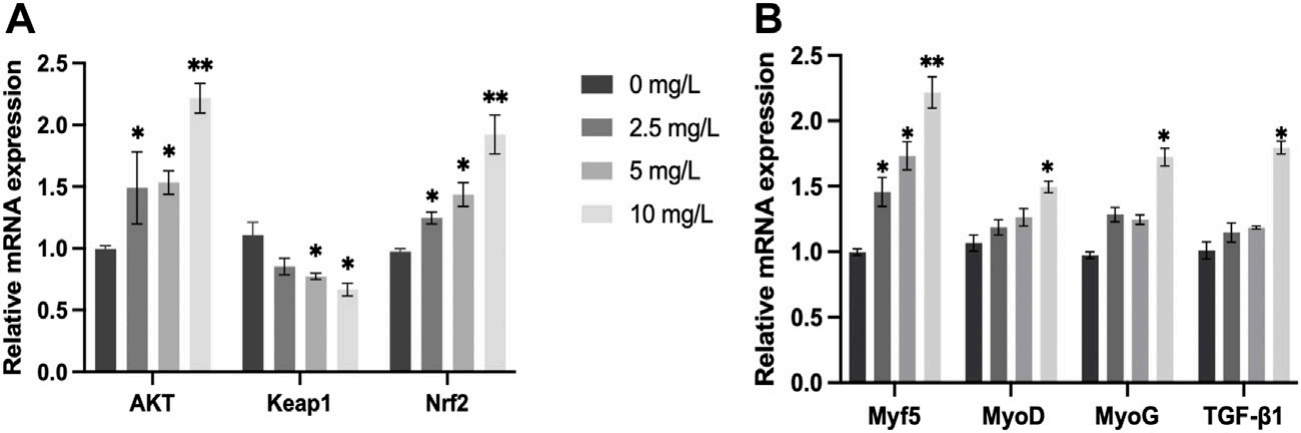
The effect of increasing concentrations of quercetin exposure on the relative expression of the **(A)**: AKT, Keap1 and Nrf2 genes and **(B)**: Myf5, MyoD, MyoG, and TGF-β1 genes in the muscle tissue of *O. potamophila* after 21 d. The bars indicate the Mean ± SD (*n* = 3). Statistical significance was taken as *(*p* < 0.05) and **(*p* < 0.01) compared with the control.

The relative mRNA expression of *AKT* and *Nrf2* increased with increasing quercetin concentrations, with significant increases in *AKT* and *Nrf2* activities at 10 mg/L quercetin (*p* < 0.01). The relative mRNA expression of *Keap1* in the muscle of *O. potamophila* decreased as concentrations of quercetin levels increased, with significant decreases at 5 mg/L and 10 mg/L quercetin (*p* < 0.05). The relative mRNA expression of *MyoD, MyoG* and *TGF-β1* increased significantly at 10 mg/L quercetin (*p* < 0.05). The relative mRNA expression of *Myf5* increased significantly at 2.5 mg/L,5 mg/L (*p* < 0.05) and 10 mg/L quercetin (*p* < 0.01) (Figure 10).

## 4 Discussion

In this study, we demonstrated changes in muscle growth-related genes and biochemical parameters after treating *O. potamophila* with quercetin resulted in the expression of. It has been shown that quercetin supplemented feed can improve the quality of lamb (Andrés et al., 2013) and chicken (Goliomytis et al., 2014) meat. *MSTN* is a member of the TGF-β superfamily and is involved in inhibiting muscle differentiation and growth. *TGF-β1* mediates the synthesis of collagen (Bradley et al., 2008). Due to the structure of MSTN, the FST complex appears to generate a complex TGF-β binding site where a TGF-β molecule can bind across a persistent electropositive gap between the two elements of FST. The gap, with a width of 60 A, allows sufficient space for the TGF-β molecules, with a length of 14–16 hexoses, analogous to that observed in the FGF growth factor acceptor heparin complex (Oelgeschläger et al., 2000). *FSTMSTN*. Two FST molecules surround the MSTN, blocking all four of its receptor binding sites, and possibly causing an antagonistic effect between FST and MSTN. the expression of *FST* in *O. potamophila* after exposure to 2.5 and 5 mg/L quercetin over 21 days increased in the intestine, muscle, and hepatopancreas and decreased in that of MSTN (a related gene responsible for controlling muscle growth) compared to the control group, further supporting the antagonistic effect of *FST* on *MSTN*. *FSTFST* The overexpression of FST in animals results in increased muscle mass, while its underexpression results in reduced muscle mass (Lee and Mcpherron, 1999). The *FST* in the brain and muscles with different somatic organizations suppressed *MSTN* expression and, as such, promoted growth in large-headed carp (Meixia et al., 2018). Myogenic regulatory factors (MRFs) related genes, as intrinsic factors affecting meat quality (Hernández-Hernández et al., 2017), have important regulatory roles in myogenic cell differentiation, muscle fiber development, and muscle tissue formation and growth (Lin et al., 2015). *MRF* is represented chronologically (Johnston et al., 2007). During somatic cell formation for carp, the first MRF family factor to be expressed is *Myf5*, followed by *MyoD* and *MEF2C*, and finally *MEF2A* (Watabe, 1999). Among them, *Myf5* and *MyoD* mainly act as myogenic determinants and *MyoG* plays an important role in myoblast differentiation (Kassar-Duchossoy et al., 2004). (Ríos et al., 2002) found experimentally that if MSTN overexpression occurs, reversible inhibition of myogenic functions can regulate myogenic fiber differentiation through downregulation of myogenin, MyoD and Myf5, and downstream creatine kinase activity. MSTN signaling specifically induces Smad 3 phosphorylation and increases Smad 3- MyoD association, suggesting that Smad 3 regulates myostatin signaling by inhibiting MyoD activity and expression (Langley et al., 2002). In the present experiment, the expression levels of *Myf5, MyoD, MyoG*, and *TGF-β1* were increased when exposed to quercetin solution. This suggests that quercetin can improve muscle formation and differentiation in *O. potamophila*. Yang (Xya et al., 2022)also found that the expression of the *TGF-β1* and *FST* genes involved in muscle growth was upregulated by ingestion of diets containing 3% HPM compared to those without, followed by improved muscle mass and increased meat firmness and chewiness. Quercetin feed supplements may therefore be able to increase muscle mass by increasing *FST, Myf5, MyoD, MyoG*, and *TGF-β1* expression while decreasing *MSTN* expression.

Quercetin reduced the levels of triglycerides and total cholesterol. APOA1 is an important component of blood lipoproteins. The principal role of APOA1 is to transport lipids and stabilize the structure of lipoproteins, which can affect liver function when impaired (Bradbury et al., 2014). Growth hormones play a role in promoting lipolysis in the body (Holder et al., 2002; Hirano et al., 2011). The relative expressions of apolipoprotein *APOA1* and the *ghra* gene in liver, muscle, and intestine tissues were lower than those in the control group, especially in the intestinal, which is due to the fact that the intestine is the primary site for lipid absorption and transport, as well as the fact that excessive quercetin concentrations are primarily concentrated in the intestine, which makes inhibition greater. Based on these findings, we conclude that quercetin functions effectively in lowering lipid levels, and maintains and promotes lipid metabolism in an organism by regulating lipid-related parameters, conserving liver function, and reducing the body-fat accumulation rate in *O. potamophila*. In general, muscle growth is usually associated with weight gain, but in this study, there was no significant weight gain over 21 days because quercetin strengthens the antioxidant capacity of the liver, strengthens genes related to lipid metabolism and affects growth, muscle growth is enhanced and lipid metabolism is also strengthened, which may be the reason for the non-gain in weight over 21 days.

To scavenge ROS, non-enzymatic and enzymatic antioxidant systems have been developed for fish (Valko et al., 2007).

GSH and CAT are important antioxidant enzymes in fish, which can scavenge hydroxyl radicals (Costantini and Verhulst, 2009), MDA is the end product of lipid peroxidation. In this study, we found that quercetin induced an increase in antioxidant enzyme activity and a decrease in MDA activity, This suggests that phosphorus can reduce lipid and protein oxidation in fish muscle. Lipid peroxidation is usually caused by ROS (Valko et al., 2007). Moreover, quercetin further mediated the mRNA expression of *AKT* and *Nrf2* by activating the PI3K/Akt/Nrf2 pathway mediated by P2X7R to improve the antioxidant capacity of the body. *Nrf2* induces the expression of protein genes that act as antioxidants and anti-inflammatory regulators and are important antioxidant genes, *Nrf2* binds to its cytoplasmic inhibitor Keap1 and is present in the cytoplasm before degradation by the proteasome (Jiang et al., 2015). Downregulation of Keap1 by quercetin treatment allows *Nrf2* to move from the cytoplasm to the nucleus to exert antioxidant effects. Upstream of *Nrf2* TOR and ribosomal S6 protein kinase1 (*S6K1*) also promote *Nrf2* expression through oxidative phosphorylation (Shay et al., 2012). PI3K/Akt signaling pathway activation promotes Nrf2 nuclear translocation, The upregulation of the *AKT* gene indicates that quercetin has an anti-apoptotic effect (Deng et al., 2013). Through the activation of Nrf2, Wang (Wang et al., 2015) found that antioxidant capacity could improve the quality of grass carp muscle and meat, water holding capacity, and tenderness. Additionally, phosphorus supplementation significantly enhances growth performance, meat and water retention in grass carp by enhancing SOD, CAT and GST activity and GSH content in grass carp (Wen et al., 2015). The enhanced antioxidant enzyme activity in fish muscle may be the result of improved transcription of antioxidant enzyme and antioxidant-related signaling molecule genes (Olsen et al., 2012; Wang et al., 2015). However, the relationship between the antioxidant mechanism of quercetin and muscle growth remains to be investigated in depth.

Feeding techniques can alter the quality of fish meat by affecting the condition of the fish and the structural and metabolic properties of the muscle tissues, resulting in changes in meat quality. Studies have shown that the juiciness and tenderness of the meat are related to its fat content and moisture, the lower the fat content the better the quality (Jeremiah et al., 1997; Rivero et al., 1999). The greater the muscle fiber density, the more tender the meat is, while muscle fiber diameter is positively correlated with tenderness. In mice fed on a quercetin supplemented diet, the reduction of muscle histopathology, the retention of muscle fiber number, and the reduction of fibrosis could result in both finer muscle fibers and greater muscle fiber density (Selsby et al., 2015). It was found that when the concentration of quercetin supplementation was higher than 5 mg/L, the muscle HDL-C and LDL-C contents were significantly lower than in the control group, that body fat was at its lowest, and body weight was relatively lower. *In vivo* studies on TG and cholesterol revealed that quercetin stimulated lipid oxidation and decreased muscle triglyceride levels (Zhang et al., 2012). The effects of quercetin supplementation discovered in this study, when taken collectively, may govern muscle growth through influencing gene expression.

## 5 Conclusion

In conclusion, full-length cDNA sequences of *FST* and *MSTN* in *O. potamophila* were obtained. The 3D structure of *FST* and *MSTN* showed that *FST* surrounded the *MSTN* ligand and blocked all four of its receptor binding sites. The biochemical parameters of muscle decreased, and quercetin was also effective in lowering lipid levels in the tissues examined. Quercetin-induced activation of *Nrf2* can upregulate several antioxidant enzymes that play important roles in combating oxidative stress. Our study provides new insights into the potential effects of quercetin supplementation on the mechanism of muscle growth and anti-oxidation properties and offers new perspectives into the potential enhancement of fish meat quality by increasing muscle fiber diameter and muscle density.

## Data Availability

The datasets presented in this study can be found in online repositories. The names of the repository/repositories and accession number(s) can be found in the article/supplementary material.
